# Supplementary material for: Causal Relationship between Aging and Anorexia Nervosa: A White-Matter-Microstructure-Mediated Mendelian Randomization Analysis
Source: Biomedicines. 2024 Aug 16;12(8):1874. doi: 10.3390/biomedicines12081874 (PMC11351342; doi:10.3390/biomedicines12081874)
Supplement: Supplementary file 1 [file biomedicines-12-01874-s001.zip › Supplementary Figure/Figure S4/Singe SNP plot of MR analysis for TL to ubm-b-1748.pdf]

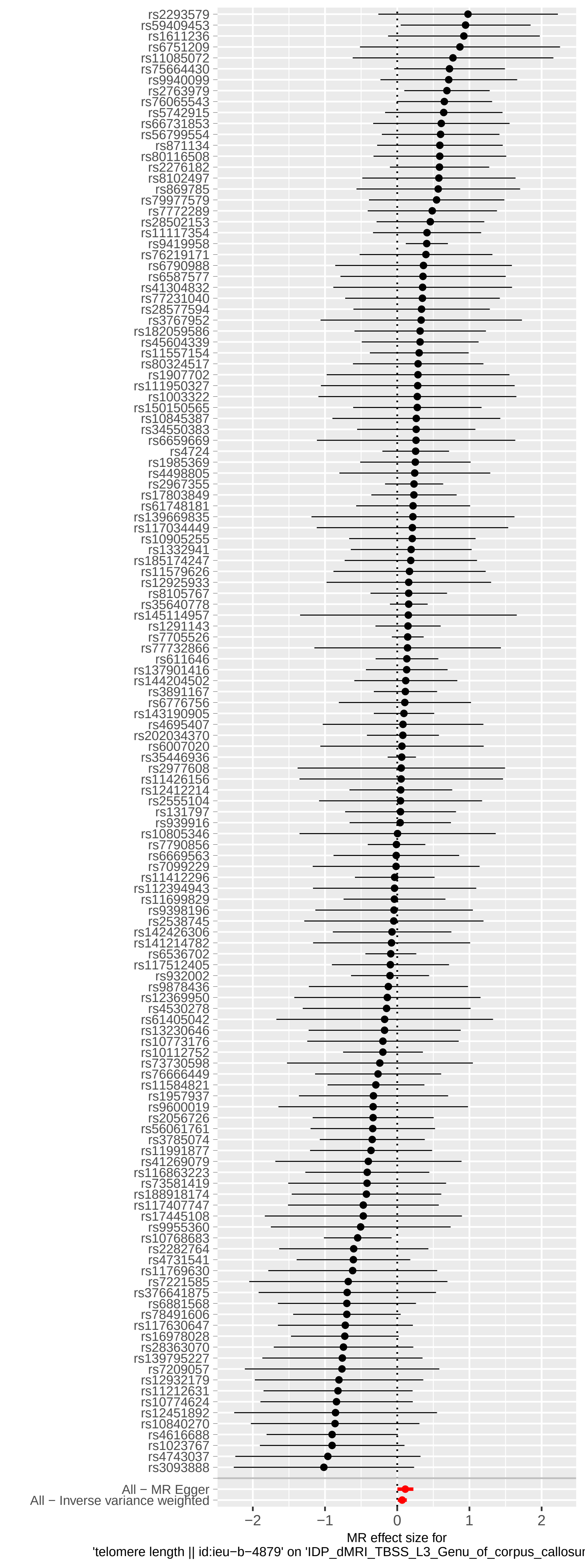

rs2293579
rs59409453
rs1611236
rs6751209
rs11085072
rs75664430
rs9940099
rs2763979
rs76065543
rs5742915
rs66731853
rs56799554
rs871134
rs80116508
rs2276182
rs8102497
rs869785
rs79977579
rs7772289
rs28502153
rs11117354
rs9419958
rs76219171
rs6790988
rs6587577
rs41304832
rs77231040
rs28577594
rs3767952
rs182059586
rs45604339
rs11557154
rs80324517
rs1907702
rs111950327
rs1003322
rs150150565
rs10845387
rs34550383
rs6659669
rs4724
rs1985369
rs4498805
rs2967355
rs17803849
rs61748181
rs139669835
rs117034449
rs10905255
rs1332941
rs185174247
rs11579626
rs12925933
rs8105767
rs35640778
rs145114957
rs1291143
rs7705526
rs77732866
rs611646
rs137901416
rs144204502
rs3891167
rs6776756
rs143190905
rs4695407
rs202034370
rs6007020
rs35446936
rs2977608
rs11426156
rs12412214
rs2555104
rs131797
rs939916
rs10805346
rs7790856
rs6669563
rs7099229
rs11412296
rs112394943
rs11699829
rs9398196
rs2538745
rs142426306
rs141214782
rs6536702
rs117512405
rs932002
rs9878436
rs12369950
rs4530278
rs61405042
rs13230646
rs10773176
rs10112752
rs73730598
rs76666449
rs11584821
rs1957937
rs9600019
rs2056726
rs56061761
rs3785074
rs11991877
rs41269079
rs116863223
rs73581419
rs188918174
rs117407747
rs17445108
rs9955360
rs10768683
rs2282764
rs4731541
rs11769630
rs7221585
rs376641875
rs6881568
rs78491606
rs117630647
rs16978028
rs28363070
rs139795227
rs7209057
rs12932179
rs11212631
rs10774624
rs12451892
rs10840270
rs4616688
rs1023767
rs4743037
rs3093888
All − MR Egger
All − Inverse variance weighted
−2
−1
0
1
2
MR effect size for
'telomere length || id:ieu−b−4879' on 'IDP_dMRI_TBSS_L3_Genu_of_corpus_callosu
